# Genetic and environmental heterogeneity of residual variance of weight traits in Nellore beef cattle

**DOI:** 10.1186/1297-9686-44-19

**Published:** 2012-07-04

**Authors:** Haroldo HR Neves, Roberto Carvalheiro, Sandra A Queiroz

**Affiliations:** 1 Departamento de Zootecnia, FCAV, UNESP, CEP: 14.884-900, Jaboticabal, SP, Brazil; 2 GenSys Consultores Associados S/S Ltda., Porto Alegre, Rio Grande do Sul, Brazil

## Abstract

**Background:**

Many studies have provided evidence of the existence of genetic heterogeneity of environmental variance, suggesting that it could be exploited to improve robustness and uniformity of livestock by selection. However, little is known about the perspectives of such a selection strategy in beef cattle.

**Methods:**

A two-step approach was applied to study the genetic heterogeneity of residual variance of weight gain from birth to weaning and long-yearling weight in a Nellore beef cattle population. First, an animal model was fitted to the data and second, the influence of additive and environmental effects on the residual variance of these traits was investigated with different models, in which the log squared estimated residuals for each phenotypic record were analyzed using the restricted maximum likelihood method. Monte Carlo simulation was performed to assess the reliability of variance component estimates from the second step and the accuracy of estimated breeding values for residual variation.

**Results:**

The results suggest that both genetic and environmental factors have an effect on the residual variance of weight gain from birth to weaning and long-yearling in Nellore beef cattle and that uniformity of these traits could be improved by selecting for lower residual variance, when considering a large amount of information to predict genetic merit for this criterion. Simulations suggested that using the two-step approach would lead to biased estimates of variance components, such that more adequate methods are needed to study the genetic heterogeneity of residual variance in beef cattle.

## Background

In most linear mixed models used in animal breeding, it is usually assumed that residual variance is constant across genotypes. However, recent studies have provided statistical support for the hypothesis that residual variance (σ²_e_) is partly under genetic control, highlighting its importance in livestock e.g. [1-4]. Comprehensive reviews on this topic have been reported in [5] and [6].

Mackay and Lyman [7] have found strong experimental evidence of genetic heterogeneity of residual variance in bristle number between isofemale lines of Drosophila. More recently, divergent selection experiments for birth weight [8] and litter size [9] in rabbits confirmed that residual variance is genetically controlled.

Such genetic differences in residual variance between individuals could be exploited to obtain more homogeneous products and/or more robust animals by selecting for lower residual variance. Although uniformity is an important issue in beef production, few studies have explored the advantages of introducing this as a selection criterion in beef cattle breeding programs. A primary concern of beef producers is to improve uniformity of production traits such as carcass weight, fat deposition and carcass composition because the price paid to producers is based on these criteria. Another interesting issue would be to increase the proportion of animals within a desired range for growth-related traits such as birth weight and mature weight.

Genetic heterogeneity of residual variance has been investigated using structural models that can estimate genetic effects on the mean and the residual variance in a single step [1-3]. A free software is also available to implement such models under a Bayesian framework [10]. However, computing time and estimability problems may hamper the use of this approach in some cases.

Rowe et al. [11] employed an alternative two-step procedure to study the genetic heterogeneity of residual variance of body weight in broilers. In the first step, body weight was analyzed with allowance for differences in residual variance among sire families, and in the second step, the resulting estimates of residual variance were analyzed by least squares methods. Another approach consists in analyzing the log squared of the estimated residuals of observations, ln(ê²) [3,8,12,13]. Mulder et al. [3] reported a method to analyze the log squared estimated residuals that is capable of estimating the genetic variance of residual variance, which gives the flexibility to account for genetic and environmental effects at the level of each record, by assuming ln(ê²) as an estimator of the residual variance, and can be implemented in standard REML packages.

Given the standard structure of beef cattle data with a relatively large number of progeny per sire and a few sibs per dam, analysis of the log squared estimated residuals should be suitable to investigate the influence of environmental and additive effects on residual variance in large populations of beef cattle. Neves et al. [14] used this approach in a exploratory work to study the genetic variability of residual variance of fifteen traits in Nellore beef cattle, but the adequacy of this method to such data structure still requires further investigation.

As suggested by Gutierrez et al. [12], this approach could be particularly interesting in an initial phase of investigation, avoiding the computational time necessary to fit high-dimensional structural models with extremely large datasets [6]. In addition, a simulation study using the unbalanced structure of a real beef cattle population may provide additional arguments in favor of the adequacy of this approach to study the genetic variability of residual variance of beef cattle traits.

Thus, the objectives of this study were: (1) to investigate the influence of additive and environmental effects on residual variance of two traits related to body weight in a large Nellore beef cattle population using a two-step approach and (2) to apply a Monte Carlo simulation of the second step, to assess the reliability of variance component estimation and the accuracy of estimated breeding values for residual variation.

## Methods

### Data

We used data from the Aliança Nelore database, comprising records from 382 Nellore herds raised with pasture systems in Brazil and Paraguay, collected from 1996 to 2009. Weight gain from birth to weaning (GBW, in kg) and long-yearling weight (YW, measured at about 550 days of age, in kg) were chosen as traits for this study. Homogeneity in GBW is an important criterion because animals with too low weights at weaning are not desired and animals with very large weights tend to have poorer performances in the subsequent period of food restriction (dry season). Likewise, more uniformity in YW would be valuable to avoid the penalties that are applied for carcass weights outside the optimum range.

Mulder et al. [3] investigated the genetic heterogeneity in residual variance of body weight in broiler chickens and fitted each sex separately, because variances in males and females differed greatly. In the present study, although the coefficients of variation were similar for both sexes, phenotypic variances were about 29% higher in males for GBW and 67% higher for YW. Since one of our objectives was to investigate the need for sex-specific analyses, heterogeneity of environmental variance was analyzed separately for each sex and the results were compared with those from a single analysis of records from both sexes.

For both traits, contemporary groups (CG) were defined based on concatenations of herd, year, and season of birth, sex, date of measurement, and management group. Genetic connectedness among CG was verified using the AMC software [15]. Briefly, this program evaluates connectedness among CG on the basis of total direct genetic links between them due to common sires, common sires and dams, or any common ancestor, in a way that genetic links are weighted by the corresponding additive relationships. A minimum of ten direct genetic links was required to include a CG in the set of connected groups that were included in the estimation of the variance components.

To reduce the impact of outliers on the estimates of variance components, only records within the range of the trait mean ± 3.5 SD were considered. In all analyses, only records for animals reared in CG with a minimum of 25 animals and sired by bulls with at least 50 progenies were considered in both steps (described in the next sections).

Information on the number of levels of fixed effects (CG), number of sires, number of dams and average progeny size of sires after data editing is presented in Table 1.

**Table 1:** Summary statistics pertaining to weight traits in Nellore beef cattle

Trait	Dataset^*^	N	N_CG_	N_S_	N_D_	N_P_	Mean (kg)	CV (%)	Skew	Kurt
GBW	males	22892	358	93	20139	246	147.48	16.11	−0.13	0.28
GBW	females	23421	373	101	20749	232	133.26	15.72	−0.10	0.35
GBW	both	51179	808	144	40485	355	140.26	17.04	−0.01	0.46
YW	males	8944	211	45	8044	199	277.49	14.64	0.44	0.34
YW	females	11915	262	64	10585	186	256.42	12.27	0.32	0.19
YW	both	28560	638	118	22154	242	278.03	14.96	0.58	0.89

GBW = weight gain from birth to weaning (in kg); YW = long-yearling weight (in kg); N = number of records per trait; N_S_ = number of sires; N_D_ = number of dams; N_CG_ = number of levels of contemporary groups; N_P_ = average size of progeny group of sires; CV = coefficient of variation (in %); Skew and Kurt = coefficients of skewness and kurtosis for the distribution of the raw data, respectively; *for each trait, analyses were carried out separately with data of males or females and also with a dataset containing records of both sexes.

### Step 1

Univariate animal models were fitted using the ASREML-R package [16], in order to estimate variance components and to obtain solutions for fixed and random effects relevant to each trait. In addition to CG, other identifiable environmental effects that significantly affected the traits in this study were considered as fixed effects. For the analysis of GBW, animal age, birth date within birth season and age of dam at calving (according to the calf’s sex) were taken in account by fitting appropriate covariates. Animal age (linear and quadratic effects) was included in the analysis of YW.

In matrix notation, the model used to analyze GBW can be represented as:

(1) $$y=Xb+Z_{1}a+Z_{2}m+Wc+e\text{,}$$

in which: **y** = vector of observations; **b** = vector of fixed effects; **a** = vector of direct additive genetic effects; **m** = vector of maternal additive genetic effects; **c** = vector of maternal permanent environmental effects; **e** = vector of residual random errors associated with observations. **X**, **Z**_**1**_, **Z**_**2**_ and **W** are incidence matrices that associate **b**, **a**, **m** and **c** to **y**, respectively. The assumptions of this model are:

(2) $$E=\left[\begin{array}{c} y\\ a\\ m\\ \begin{array}{l} c\\ e \end{array} \end{array}\right]=\left[\begin{array}{c} Xb\\ 0\\ 0\\ \begin{array}{l} 0\\ 0 \end{array} \end{array}\right];V\left[\begin{array}{c} a\\ m\\ c\\ e \end{array}\right]=\left[\begin{array}{ccc} G⊗A & 0 & 0\\ 0 & I_{d}⊗\sigma^{2}{}_{c} & 0\\ 0 & 0 & I_{n}⊗R \end{array}\right]$$

where: **G** = matrix of genetic covariances between direct and maternal effects, **A** = numerator relationship matrix, σ²_c_ = maternal permanent environmental variance, **I**_**d**_ = identity matrix of order equal to the number of dams, **I**_**n**_ = identity matrix of order equal to the number of observations, **R** = residual (co) variance matrix. It was assumed that $G=\left[\begin{array}{cc} \sigma^{2}{}_{a} & \sigma_{\mathrm{am}}\\ \sigma_{\mathrm{am}} & \sigma^{2}{}_{m} \end{array}\right]$, where *σ²*_*a*_ = variance of direct additive genetic effects, *σ²*_*m*_ = variance of maternal additive genetic effects, and *σ*_*am*_ = covariance between maternal and direct additive genetic effects. Since only a few dams had more than one offspring of each sex with information on GBW, the maternal permanent environmental effect was not included when the information of each sex was considered separately because the data structure would not allow adequate estimation of this parameter. Also due to the data structure, *σ*_*am*_ was set to zero in all analyses (most dams had no data for their own performance and a large number of sires had no maternal grandprogeny records).

In order to estimate the effect of allowing for heterogeneous residual variances in the first step, a first set of analyses was carried out by assuming a homoscedastic model (HOM). Then, an equivalent set of analyses was carried out, allowing for different residual variances for each sire family (model HET). Therefore, matrix **R** of equation 1 is a diagonal matrix, in which the elements are either equal to σ²_e_ (model HOM) or equal to σ²_ej_ (model HET), where σ²_ej_ is the residual variance specific to the progeny of the j^th^ sire.

The model used to analyze the YW trait included only direct additive effects and residuals as random effects. This model was chosen because including both types of maternal effects described in equation (1) did not improve model fitting (evaluated by the statistics Akaike Information Criterion (AIC) and the Bayesian Information Criterion (BIC)).

Thus, variance components for GBW and YW were estimated by considering different sources of data (i.e. data from males, females or both sexes) and different assumptions on residual variance in the first step (i.e. HOM or HET model). In addition, 10-fold cross-validation was performed to investigate the predictive ability of the HOM and HET models for each analyzed dataset. In this case, each dataset was randomly partitioned into 10 disjoint subsets, each with approximately one tenth of the records. In each case, nine of the subsets were used for model fitting (training set), after which the observations in the remaining subset were predicted (testing set) and the mean squared error (MSE) of prediction was calculated. At the end of this process, the average of the MSE was calculated, with the estimates of MSE obtained separately for each testing set.

### Step 2

Solutions obtained in the first step were used to estimate the residual (ê) for each observation. For each trait, log squared estimated residuals, ln(ê²), were used as a measure of the residual variance, as discussed previously.

In order to investigate the influence of fixed effects (CG) and additive genetic effects on the residual variance of GBW and YW, different models were fitted to the log squared estimated residuals in ASREML-R. The full model of the second step can be represented by:

(3) $$\ln({\hat{\text{e}}}^{2})=Xb^{*}+Z_{1}a^{*}+Z_{2}m^{*}+\epsilon \text{,}$$

where **ln(ê²)** = vector of individual log squared estimated residuals for the trait analyzed; b^*^ = vector of fixed effects; a^*^ = vector of direct additive genetic effects; m^*^ = vector of maternal additive genetic effects, and **ϵ** = vector of residual random errors associated with the log squared estimated residuals. Assumptions of model (2) were:

(4) $$E=\left[\begin{array}{c} \ln\left({\hat{e}}^{2}\right)\\ a^{*}\\ m^{*}\\ \epsilon \end{array}\right]=\left[\begin{array}{c} Xb^{*}\\ 0\\ 0\\ 0 \end{array}\right];V=\left[\begin{array}{c} a^{*}\\ m^{*}\\ \epsilon \end{array}\right]=\left[\begin{array}{cc} G^{*}⊗A & 0\\ 0 & I_{n}⊗R^{*} \end{array}\right]$$

where: G^*^ = matrix of genetic covariances, **A** = numerator relationship matrix, **I**_**n**_ = identity matrix of order equal to the number of observations, R^*^ = residual variance (σ²ϵ).

It was assumed that $G^{*}=\left[\begin{array}{cc} \sigma^{2}{}_{Av,\exp} & 0\\ 0 & \sigma^{2}{}_{m,\exp} \end{array}\right]$, where *σ²*_*Av,exp*_ is the variance due to direct additive genetic effects and *σ²*_*m,exp*_ is the variance due to maternal additive genetic effects on the log squared estimated residuals. As in the first step, maternal effects only improved model fitting for GBW (according to AIC and BIC) and thus were not considered in the case of YW.

Results from model (2) and reduced models were compared to evaluate the importance of including direct additive genetic effects and maternal additive effects in the analyses of the log squared estimated residuals. A likelihood-ratio test was applied to assess the significance of considering additive genetic effects at the level of residual variance. The statistics BIC and AIC were also calculated, in order to penalize models with a larger number of parameters.

### Genetic parameters for residual variation

Two parameters, namely evolvability of residual variance (Ev) and heritability of residual variance (h²_v_) were calculated in order to measure the strength of genetic variation on residual variance and also to compare our results with those of previous studies. Because the additive variance estimates (σ²_Av,exp_) from the second step were expressed on an exponential scale, they were transformed as proposed by [3] into estimates on the scale of residual variance (i.e. *σ²*_*AV*_), which were used to compute Ev.

Evolvability was proposed by Houle [17] to quantify the possibility of changing the mean of a trait by selection. In the present context, Ev reflects the opportunity to change the average residual variance by selection. Ev was calculated as an additive coefficient of variation (Ev = *σ*_*AV*_*/ σ²*_*ê*_). Parameter σ²_ê_ is an estimate of the residual variance and was equal to the REML estimate of residual variance for the HOM model and the average of residual variance among sire families for the HET model.

The heritability of residual variance (h²_v_) was derived by [5] as a regression coefficient of breeding values for residual variance (*A*_*v*_) on squared phenotypes and it has been used as a central parameter in calculation of the accuracy of predicted breeding values for residual variance (r_*Av,Âv*_). Here, h²v was calculated as reported in [3], while the heritability of ln(ê²) and its standard error (s.e.) were directly estimated on a log scale and were used to approximate the standard error of h²_v_.

The Pearson’s correlation coefficient between estimated breeding values (EBV) of sires for the mean and for log squared estimated residuals was calculated to provide a rough indication of the genetic association between these criteria, since direct estimation of this parameter is not feasible with the two-step approach. Because this estimate is dependent on the accuracy of EBV, only sires with more than 50 progeny were used, in order to reduce this source of bias.

### Simulation study

#### Observed pedigree

The data structure of the population described previously was used to simulate trait Y. Twenty replicates were simulated for each scenario described below. We considered animals of both sexes with information on YW (Table 1).

For individuals with records, trait Y, expressed in kg, was simulated as follows:

(5) $$y_{\mathrm{ij}}=b_{\mathrm{mi}}+am_{\mathrm{ij}}+\exp((b_{\mathrm{vi}}+av_{\mathrm{ij}})/2)\epsilon_{\mathrm{ij}}\text{,}$$

where *b*_*mi*_ is the fixed effect simulated for the i^th^ CG, *am*_ij_ is the breeding value of animal j for the mean of Y and the last term replicates random residual effects according to the exponential model described by SanCristóbal-Gaudy et al. [1]. At the level of the log of residual variance, we simulated a fixed effect due to the i^th^ CG (*b*_*vi*_), the breeding value of j^th^ animal (*av*_*ij*_) and *ϵ*_*ij*_ is independent and identically distributed as N (0;1).

With the aim of mimicking the real effects of CG on the mean and the residual variance of Y, CG effects b_m_ were drawn from a uniform distribution defined by U(300;400) and values for b_v_ were drawn from a uniform distribution U(5.40;5.80).

For the founder animals, *am* and *av* were simulated from a **MVN(0**,**G**_**mv**_**)**, in which $G_{\mathrm{mv}}=\left[\begin{array}{cc} \sigma^{2}{}_{a} & \sigma_{\mathrm{mv}}\\ \sigma_{\mathrm{mv}} & \sigma^{2}{}_{Av,\exp} \end{array}\right]$, with σ*²*_*a*_ = 200 and *σ²*_*Av,exp*_ equal to 0.01, 0.09 or 0.25. The covariance between direct additive genetic effects on the mean and the residual variance (*σ*_*mv*_*)* was simulated such that three scenarios for the additive genetic correlation between the mean and the residual variance of Y could be investigated (r_mv_ = −0.50; 0 or 0.50).

For subsequent generations, breeding values of non-founder animals were simulated as the average of the breeding values of the sire and dam plus a random Mendelian sampling term.

Accordingly, the expected value of the environmental variance (σ²_e_) is equal to exp(*b*_*v*_)*exp(σ²_*Av,exp*_/2). Thus, Y was simulated to resemble a trait with heritability around 0.40, a mean equal to 350 kg and a phenotypic variance of about 480 kg² (because the value simulated for *σ²*_*Av,exp*_ affects the expectation of σ²_e_ and the values for heritability and phenotypic variance vary depending on *σ²*_*Av,exp*_ ). According to formulas in [3], the parameters simulated at the level of the log residual variance, when converted to the scale of the residual variance, result in Ev equal to about 10%, 30% and 50%, depending on the value simulated for *σ²*_*Av,exp*_ (0.01,0.09 or 0.25, respectively) .

The two-step approach described in the previous section was used to analyze each replicate of the simulated data. In the first step, an animal model was fitted, including CG as fixed effect, considering two scenarios for residual variance HOM or HET. In the second step, models with the same definition as equation (2) were employed, except that maternal additive effects were not included. Estimates of *σ²*_*Av,exp*_ according to each model were compared with corresponding true values. The accuracy of prediction of breeding values for log squared estimated residuals was assessed with the Pearson’s correlation between estimated and true breeding values, by considering only sires, only dams and all animals in the pedigree.

#### Simulated pedigree

In order to investigate the adequacy of the methods proposed here with a more balanced dataset, the same simulation parameters as described in the previous section were used to simulate five generations of an artificial population with 25 000 records. The base population consisted in 5 000 dams and 100 sires. At each generation, sires (dams) had 50 (1) offspring and records were distributed across 100 CG, such that each CG included the progeny of five sires, each with 10 sons. The simulation process considered a 20% replacement rate for both sexes per generation, assuming random mating and no selection. Data were analyzed and results were compared in the same way as described in the previous section.

## Results

### Step 1

Summary statistics of data for GBW and YW are presented in Table 1. The values for skewness and kurtosis indicate an approximate normal distribution of data for GBW and a slightly positively skewed distribution in the case of YW (Table 1), for both males and females.

Although the estimates of variance components were very similar for models HOM and HET for both GBW and YW, model HET had a better fit according to AIC, while model HOM provided a better fit according to BIC (Table 2). As a general rule, model HOM had slightly lower average MSE than model HET, which indicates that model HOM has a higher predictive ability. The only exception was for GBW in the dataset with female records, for which model HET performed slightly better in terms of predictive ability.

**Table 2:** Results of variance component estimation according to trait, sex^*^and residual variance structure^**^

Trait/Sex	Model	σ²_a_	σ²_e_	h²_d_	σ²_m_	σ²_c_	AIC	BIC	MSE
GBW/M	HOM	44 (9)	226 (8)	0.12 (0.02)	113 (7)		157113	157137	380.54
GBW/M	HET	44 (8)	225 (8)	0.11 (0.02)	120 (7)		156986	157748	381.22
GBW/F	HOM	31 (6)	193 (6)	0.10 (0.02)	78 (5)		155334	155358	305.67
GBW/F	HET	31 (6)	192 (7)	0.10 (0.02)	84 (5)		155163	155992	305.51
GBW/B	HOM	44 (7)	209 (4)	0.12 (0.02)	53 (12)	47 (12)	346263	346298	337.94
GBW/B	HET	42 (6)	212 (7)	0.12 (0.02)	58 (12)	47 (12)	345807	347105	338.17
YW/M	HOM	308 (58)	292 (44)	0.51 (0.08)			64302	64316	569.00
YW/M	HET	312 (58)	268 (18)	0.54 (0.05)			64267	64593	569.19
YW/F	HOM	177 (24)	281 (18)	0.39 (0.05)			82970	82985	439.54
YW/F	HET	183 (24)	279 (12)	0.40 (0.03)			82907	83386	439.97
YW/B	HOM	220 (15)	310 (11)	0.42 (0.03)			202426	202443	499.54
YW/B	HET	218 (15)	306 (10)	0.42 (0.02)			202249	203229	500.04

^*^GBW = weight gain from birth to weaning; YW = long-yearling weight; M = males; F = females; B = both sexes; ^**^HOM = model with homogeneous residual variance; HET = model allowing for differences between sire families in residual variance; σ²_a_ = estimated variance for additive direct genetic effect for the mean of each trait; σ²_e_ = estimated residual variance (average residual variance among sire families), in the case of model HOM (HET); σ²_m_ = estimated variance due to maternal additive genetic effects for the mean of GBW; σ²_c_ = estimated variance due to maternal permanent environmental effects for the mean of GBW; AIC = Akaike Information Criterion; BIC = Bayesian Information Criterion. MSE = average mean squared error of prediction based on a 10-fold cross-validation; standard errors are presented between brackets.

When each sex was analyzed separately, estimates of direct heritability (h²) were higher for males than for females (about 12% and 33% for GBW and YW, respectively) for both models HOM and HET. Higher estimates of average residual variance and genetic additive (direct and maternal) variance were also obtained for males than for females, possibly because of scale effects. When the data on males and females were considered jointly, the estimates of h² (s.e.) were similar for models HOM and HET, i.e. about 0.12 (0.02) and 0.41 (0.03) for GBW and YW, respectively.

### Step 2

If the residuals from the first step are normally distributed, their squares will follow a χ² distribution [6]. For both GBW and YW, the distributions of the log squared residuals were close to the log of a chi-squared variable, (skewness about −1.5 and excess kurtosis about 1.0), regardless of whether model HET or HOM was fitted in the first step (data not shown), and whether female or male records were considered.

Estimates of variance components and related parameters obtained from the second step are presented in Table 3, as well as the results for information criteria. When a heteroscedastic model was fitted in the first step (HET), results of likelihood-ratio tests indicated that fitting additive genetic effects at the level of the (log) residual variance significantly improved model fitting (P < 0.0001), compared to a situation in which only fixed effects were considered, for both GBW and YW and regardless of the source of information (males, females, or both). The same conclusion holds when such models are compared using information criteria (AIC and BIC, Table 3).

**Table 3:** Estimates of parameters related to the residual variance of weight traits in Nellore beef cattle

Trait/Sex^*^	Model^**^	*σ²*_Av*,exp*_	*σ²*_m*,exp*_	dAIC	dBIC	*σ²*_Av_	Ev(%)	h²_v_(%)	r_mv_
GBW/M	HOM	0.0165	0.549	0.19	8.2	372	8.54	0.13	0.23
		(0.02)	(0.09)						
GBW/M	HET	1.07	0.297	−276	−285	21500	65.20	5.87	0.21
		(0.19)	(0.11)						
GBW/F	HOM	2E-7	0.105	2	10	0.00	0.03	0.00	0.12
		(0.00)	(0.08)						
GBW/F	HET	0.495	0.0614	−121	−129	7030	43.80	3.37	0.26
		(0.10)	(0.09)						
GBW/B	HOM	0.0404	0.265	−16	−7.1	730	12.90	0.39	0.27
		(0.02)	(0.04)						
GBW/B	HET	0.476	0.189	−350	−340	8570	43.70	3.89	0.27
		(0.07)	(0.05)						
YW/M	HOM	2E-7		2	9.1	0.01	0.03	0.00	0.12
		(0.00)							
YW/M	HET	1.68		−240	−230	44400	78.70	5.50	−0.01
		(0.32)							
YW/F	HOM	0.0277		0.83	8.2	881	10.60	0.21	0.21
		(0.03)							
YW/F	HET	0.457		−79	−71	14200	42.70	3.02	0.36
		(0.11)							
YW/B	HOM	0.0193		−0.002	8.2	740	8.77	0.13	0.25
		(0.02)							
YW/B	HET	0.473		−195	−187	17300	43.00	2.89	0.23
		(0.08)							

^*^GBW = weight gain from birth to weaning; YW = long-yearling weight; M = males; F = females; B = both sexes; ^**^HOM = assuming homogeneous residual variance in model (1); HET = model allowing for differences between sire families in residual variance. *σ²*_Av*,exp*_*:* estimated additive genetic variance for log squared of estimated residuals, ln(ê²); *σ²*_m,exp_*:* estimated maternal additive genetic variance for ln(ê²); *σ²*_Av_:estimates of additive genetic variance on the scale of the residual variance (σ²_e_), assuming the quantitative model for genetic heterogeneity of σ²_e_[5]; Ev: evolvability of σ²_e_ in %; h²_v_: heritability of σ²_e_; r_mv_: Pearson’s correlation between sire EBV for mean and for σ²_e_; AIC = Akaike Information Criterion; BIC = Bayesian Information Criterion; dAIC (dBIC): difference between the AIC (BIC) obtained for a model considering additive genetic effects on ln(ê²) and the AIC (BIC) of a model only with fixed effects; negative values indicate that better fit was obtained with the model considering genetic heterogeneity on ln(ê²); standard errors are presented between brackets.

Conversely, the influence of additive genetic effects on residual variance was weaker when model HOM was fitted in the first step. Information criteria did not favor the model with additive effects on residual variance for YW, regardless of the source of information (males, females, or both), while these criteria only favored the model with additive genetic effects on the residual variation of GBW when records of both males and females were analyzed jointly (GBW/B, Table 3).

For both traits, estimates of additive variance for the log squared estimated residuals from model HET were at least 12-fold larger than comparable estimates from model HOM (Table 3). In addition, estimates of *σ²*_Av*,exp*_ obtained after fitting model HOM in the first step were very close to zero and, given the magnitude of their standard errors, they did not provide reasonable evidence of additive variance on ln(ê²). For all traits, regardless of the animal’s sex, a 95% confidence interval for *σ²*_Av*,exp*_ did not include zero when the first step accounted for differences between sire families in residual variance.

In the case of GBW, taking into account both direct and maternal additive effects improved the model fitting in the second step. When model HOM was fitted in the first step, estimates of the maternal additive effect for ln(ê²) were between 40% and 80% larger than those obtained when model HET was fitted in the first step.

Under model HET, *σ²*_Av*,exp*_ estimates were at least two-fold higher in males than in females. For a same trait, the *σ²*_Av*,exp*_ estimated for females was close to that obtained when records of both sexes were analyzed jointly (Table 3).

Estimates of Ev were less than 13% in all situations when model HOM was fitted in the first step but were as high as 65% and 78% for GBW and YW, respectively, when only the data from males were considered and model HET was fitted in the first step. Interestingly, for both GBW and YW, Ev estimates were around 43% in situations when only records of females were considered and also when records of both sexes were combined (Table 3).

Values of the heritability of residual variance (h²_v_) were always less than 0.5% under model HOM, while they ranged from 3 to 6% when model HET was fitted. Again, h²_v_ estimates were higher in males than in females, for both GBW and YW. The approximated standard error of h²_v_ was small when model HET was fitted in the first step (data not shown), such that the 95% confidence interval did not include zero.

In general, the Pearson’s correlation between the sire’s EBV for the mean and for the log squared estimated residuals was positive and low (below 0.40). The only exception was for YW in males, for which the estimated correlation was very close to zero (Table 3).

### Simulation study

For both sets of simulations (using observed or simulated pedigrees), the estimated variance components for the mean of the simulated trait were in reasonable agreement with the simulated (true) values, such that the absolute values of the relative bias for estimates of additive variance and residual variance were at most 3.7% and 1.7%, respectively (data not shown). Also, there was no evidence that fitting model HET instead of model HOM in the first step resulted in estimates of residual variance closer to the simulated values, for all scenarios of additive variance, residual variance and correlation between mean and residual variance.

In the simulations using the observed pedigree and for all scenarios, estimates of additive variance obtained in the second step were biased. If model HOM was fitted in the first step, the estimates of *σ²*_Av*,exp*_ were biased downwards (between 35%–60% lower than the true values) (Table 4) and if model HET was fitted in the first step, they were biased upwards, although the amount of relative bias was larger for model HET, in all scenarios.

**Table 4:** **Bias in estimation of *σ²***_**Av*,exp***_ and correlation between mean and residual variance for a simulated trait^*^

Observed pedigree							
Model	r_mv_	***σ²***_Av*,exp*_ = 0.01		***σ²***_Av***,exp***_ = 0.09		***σ²***_Av***,exp***_ = 0.25	
		Bias (%)	rˆ_mv_	Bias (%)	rˆ_mv_	Bias (%)	rˆ_mv_
HOM	−0.5	−37.2	−0.13	−55.4	−0.26	−58.5	−0.32
		(16.2)	(0.03)	(5.2)	(0.02)	(2.7)	(0.02)
HET	−0.5	1842.4	−0.15	302.8	−0.29	169.9	−0.35
		(101.5)	(0.02)	(20.2)	(0.02)	(51.8)	(0.02)
HOM	0	−35.4	0.02	−53.7	0.01	−55.1	0.02
		(12.9)	(0.02)	(5.2)	(0.02)	(3.3)	(0.02)
HET	0	1957.8	0.00	291.2	0.01	142.4	0.02
		(160.3)	(0.02)	(17.3)	(0.02)	(10.1)	(0.02)
HOM	0.5	−44.5	0.11	−61.9	0.21	−56.4	0.30
		(18.1)	(0.02)	(4.8)	(0.02)	(2.7)	(0.02)
HET	0.5	1820.0	0.13	311.4	0.26	131.5	0.34
		(184.4)	(0.02)	(24.8)	(0.02)	(8.9)	(0.02)
Simulated pedigree							
**Model**	**r**_**mv**_	***σ²***_**Av*,exp***_** = 0.01**		***σ²***_***Av,exp***_*** = 0.09***		***σ²***_***Av,exp***_*** = 0.25***	
		**Bias (%)**	**rˆ**_**mv**_	**Bias (%)**	**rˆ**_**mv**_	**Bias (%)**	**rˆ**_**mv**_
HOM	−0.5	68.30	−0.05	−45.74	−0.20	−53.37	−0.26
		(40.80)	(0.02)	(5.62)	(0.01)	(3.35)	(0.02)
HET	−0.5	2993.04	−0.08	389.63	−0.23	133.73	−0.30
		(156.76)	(0.02)	(18.90)	(0.01)	(13.85)	(0.02)
HOM	0	56.29	−0.03	−51.58	−0.02	−50.35	0.01
		(42.09)	(0.02)	(5.07)	(0.02)	(3.46)	(0.02)
HET	0	3062.76	0.02	361.38	0.01	136.38	−0.02
		(131.99)	(0.02)	(18.28)	(0.01)	(7.71)	(0.02)
HOM	0.5	11.47	0.05	−47.80	0.21	−52.36	0.27
		(34.49)	(0.02)	(5.99)	(0.01)	(3.90)	(0.01)
HET	0.5	2684.63	0.08	363.39	0.23	108.27	0.31
		(184.43)	(0.01)	(19.62)	(0.01)	(16.81)	(0.01)

^*^ Results obtained after analyses of log squared estimated residuals under different scenarios, given as mean (s.e.) of 20 replicates. Each scenario is characterized by the simulated values for additive genetic variance on residual variance (*σ²*_Av*,exp*_), according to the model postulated by [15] and for the additive genetic correlation between the mean and the residual variance of the trait. Homoscedastic (HOM) or heterogeneous residual variances among sire families (HET) were assumed in the model for the mean of the trait (first step of analyses); simulations employed either the observed pedigree structure or a simulated pedigree; bias (%) = relative bias, computed as deviation between the estimated and the simulated value for *σ²*_Av*,exp*_, as percentage of the simulated parameter; r_mv =_ simulated value for additive genetic correlation between the mean and the residual variance of the simulated trait; rˆ_mv =_ Pearson’s correlation between sire EBV for mean and for residual variance.

With a more balanced design (simulated pedigree), the amount of bias in estimates from the second step was similar to that obtained in the simulations using the observed pedigree (Table 4). The only major difference was found for model HOM and if the true value of *σ²*_Av*,exp*_ was equal to 0.01. In this case, *σ²*_Av*,exp*_ was underestimated in the simulation using the observed pedigree, while it was overestimated in the simulation using the simulated pedigree.

For both sets of simulations, when heterogeneous residual variances among sire families were accounted for in the first step, the higher the simulated values for *σ²*_Av*,exp*_, the lower the amount of relative bias, which was not observed for model HOM (Table 4). It should be noted that when a small value was simulated for *σ²*_Av*,exp*_ (i.e. 0.01), this parameter was overestimated almost 20-fold (30-fold) under model HET compared to the true value in the case of simulations using the observed (simulated) pedigree. Similarly, the higher the values simulated for *σ²*_Av*,exp*_, the closer the agreement between the true additive genetic correlation between mean and residual variance and the estimates based on Pearson’s correlation between estimated breeding values for these criteria (rˆ_mv_), regardless of the assumptions about residual variance in the first step.

In the simulations using the observed pedigree, average accuracies of predicted breeding values for the log squared estimated residuals were in reasonable agreement with the expected values calculated according to formulas in [3], given the amount of information available and the values simulated for the heritability of residual variance (h²_v_). In general, meaningful values (above 0.50) were observed only for accuracies averaged across all sires when *σ²*_Av*,exp*_ was greater than 0.09 (Table 5). Regardless of the value simulated for the correlation between mean and residual variance, the highest average accuracy was about 0.30 when considering the EBV of all animals and around 0.15 when considering only dams, which was for the scenario in which *σ²*_Av*,exp*_ was equal to 0.25. The values obtained in the simulation using the simulated pedigree were very similar to those obtained in the simulation using the observed pedigree and are therefore not presented.

**Table 5:** Average accuracy of prediction of breeding values for log squared estimated residuals for a simulated trait^*^

		***σ²***_**Av*,exp***_** = 0.01**			***σ²***_**Av*,exp***_** = 0.09**			***σ²***_**Av*,exp***_** = 0.25**		
Model	r_mv_	All	Sire	Dam	All	Sire	Dam	All	Sire	Dam
HOM	−0.5	0.11	0.20	0.05	0.25	0.50	0.12	0.29	0.63	0.14
		(0.02)	(0.02)	(0.01)	(0.01)	(0.01)	(0.00)	(0.01)	(0.01)	(0.00)
HET	−0.5	0.11	0.23	0.05	0.26	0.57	0.11	0.30	0.70	0.14
		(0.01)	(0.02)	(0.00)	(0.01)	(0.02)	(0.00)	(0.01)	(0.02)	(0.00)
HOM	0	0.16	0.20	0.07	0.26	0.47	0.12	0.31	0.64	0.15
		(0.02)	(0.02)	(0.01)	(0.02)	(0.01)	(0.01)	(0.01)	(0.02)	(0.01)
HET	0	0.13	0.22	0.06	0.27	0.55	0.12	0.31	0.71	0.15
		(0.01)	(0.02)	(0.01)	(0.02)	(0.01)	(0.01)	(0.01)	(0.01)	(0.01)
HOM	0.5	0.12	0.21	0.05	0.22	0.44	0.10	0.30	0.63	0.15
		(0.02)	(0.02)	(0.01)	(0.02)	(0.02)	(0.01)	(0.01)	(0.01)	(0.00)
HET	0.5	0.12	0.23	0.05	0.23	0.53	0.10	0.30	0.70	0.14
		(0.01)	(0.02)	(0.00)	(0.01)	(0.01)	(0.00)	(0.01)	(0.01)	(0.00)

^*^Results given as mean (s.e.) of 20 replicates; accuracies were computed as Pearson’s correlations between estimated and true breeding values, by considering only the sires (sire), only the dams (dam) or all animals in the pedigree (all); results pertain to simulation using the observed pedigree structure; each scenario is characterized by the simulated values for additive genetic variance on residual variance (*σ²*_Av*,exp*_) and for the additive genetic correlation between the mean and the residual variance of the trait (r_mv_); homoscedastic (HOM) or heterogeneous residual variances among sire families (HET) were assumed in the model for the mean of the trait (first step of analyses).

In general, comparing the accuracies obtained when model HET versus HOM was fitted in the first step shows that allowing for heterogeneous residual variances in the first step resulted in higher accuracies of predicted EBV for ln(ê²) for sires (between 8% and 20% higher) (Table 5).

## Discussion

### Genetic parameters for the mean

Parameters associated with additive genetic effects on GBW and YW means obtained after the first step of this study are consistent with results of previous studies reporting low to moderate estimates of heritability for direct and maternal effects on pre-weaning growth traits [18,19], and moderate to high values for YW [20,21] in Nellore cattle.

When analyses were carried out separately for each sex, heritability estimates were higher in males, for both GBW and YW (notably in the case of YW). Conversely, heritability estimates were higher in females for yearling weight gain in Holstein cattle [22], body weight in broiler chickens [3,23], and slaughter weight in pigs [4] and such discrepancies deserve further investigation.

### Genetic parameters for the residual variance

Results from the second step showed stronger evidence for genetic heterogeneity of residual variance if heterogeneous residual variances were allowed for in the first step, although more extensive comparisons could not be made based on restricted likelihood, e.g. comparison of models that assume different combinations of fixed effects on residual variance.

Similar to results reported by [3], it is not clear whether fitting a model with heterogeneous residual variances among sire families in the first step provides a better fit, for both GBW and YW. Although AIC favored model HET, support for the HET model was weaker than for the HOM model when a more severe penalty for complexity (BIC) was applied. In addition, model HOM had a slightly higher predictive ability. This lack of agreement between AIC and BIC could indicate that residual variances are estimated with low precision, rather than weak evidence for genetic heterogeneity of residual variance [3]. Based on this argument, imposing a minimum progeny size for sires to be considered in the analysis seemed a natural choice. However, given the typical data structure of beef cattle populations, imposing this type of restriction would make it more difficult to obtain a representative sample of sire families, especially if additional restrictions with respect to the time period in which data are collected are made.

Genetic parameters for residual variance in this same population were estimated in an exploratory study [14], in which model HOM was fitted for 15 traits considering records on both sexes and most estimates of Ev were between 15 and 25%. In the present study, estimates of Ev for comparable models were lower (about 13% and 9% for GBW and YW, respectively), which may be explained by the fact that the records considered cover a shorter period of time than in [14].

Despite this, results of both studies suggest that genetic additive effects act on the residual variance, while there is reasonable evidence of both genetic and environmental heterogeneity of residual variance for traits related to body weight in other livestock species, such as broilers [3,11,23], rabbits [8] and pigs [4].

The estimated Ev under model HET were more consistent with previous estimates for body weight in other species [5,6], while model HOM provided lower estimates. In broilers, [3] and [23] applied approaches similar to those in this study and reported Ev estimates ranging between 35 and 55%, depending on the animal’s sex, while [11] reported coefficients around 30%, but with a different approach. In pigs, estimates of Ev were about 34% for body weight at slaughter [4] and in rabbits, about 25% for birth weight [8]. The low h²v estimates obtained for both GBW and YW are in agreement with value ranges reported in previous studies [5,6], which means that a large amount of information is needed to predict accurately the EBV for residual variance.

Nevertheless, given the magnitude of the estimated Ev for weight gain from birth to weaning and long-yearling weight, when model HET was fitted in the first step, a meaningful response to selection on EBV for residual variance of these traits would be expected in the case of large sire families.

Results from other studies also indicate that selection on EBV for residual variance can improve uniformity. In broilers, [3] predicted reductions up to 20–30% in residual variance of body weight after one generation when selecting only on this criterion. Empirical evidence for this approach comes from the divergent selection experiment reported by Garreau et al. [8], in which, after four generations of selection there was a difference of 27.3% between lines in within-litter variance of birth weight in rabbits.

Considerable differences between males and females were observed in genetic parameters for residual variation. Similar results were also reported by [3], which suggested that residual variance has a different genetic basis in each sex, such that using sex-specific variance ratios could be valuable to estimate breeding values. It was expected that, when data of both sexes were analyzed jointly, the estimates for Ev and h²v would be intermediate between those obtained for each sex, however in the present study, the resulting estimates were very close to those obtained for females under model HET.

Although this question deserves further investigation, using a well-designed dataset, implementing sex-specific analyses is not possible in situations in which the number of progeny of each sex is small and the data structure does not permit adequate estimation of effects at the level of residual variance. For instance, due to the restrictions imposed on the size of contemporary groups and progeny groups, a substantial amount of data could not be considered in sex-specific analyses (about 10% and 27% of the records available for GBW and YW, respectively).

### Genetic relationship between mean and residual variance

An important issue in selection for residual variance is the genetic association between mean and residual variance (r_mv_). Since direct estimation of r_mv_ was not feasible under the current approach, the correlation between sire EBV for mean and residual variance (rˆ_mv_) was used as an indicator of the direction of this association, as also applied by [23]. Simulations suggested that the correlation between breeding values for mean and log squared estimated residuals is consistent with the sign of the true correlation, but the quality of this estimate as an approximation of r_mv_ depended on the magnitude of the genetic variance of residual variance.

As a general rule, estimates of r_mv_ were moderate and positive for both GBW and YW. While some studies have reported negative genetic correlations between mean and residual variance [3,11,12,23], other authors have obtained positive values for this correlation [12,24] for traits related to body weight in different species.

### Adequacy of the two-step approach

The two-step approach applied in the present study made it possible to estimate the genetic variance of residual variance in a relatively large population, but the simulations suggested that the investigated models provide biased estimates of genetic parameters for residual variance. The similar pattern of bias found for simulations with simulated and observed pedigrees suggests that this bias may be caused much more by the nature of the two-step approach than by data structure.

As a general rule, model HOM resulted in underestimated genetic variance of residual variance, while it was overestimated when the first step accounted for heterogeneous residual variances among sire families (model HET). Thus, it is expected that true values for genetic variance of residual variance of GBW and YW are somewhat intermediate between those obtained under models HOM and HET.

Although the amount of bias was higher when model HET rather than model HOM was fitted in the first step, the accuracies of prediction were also higher for model HET. These findings must be interpreted with caution since the environmental noise simulated at the level of the residual variance was only due to a fixed effect (CG), which is probably a situation more favorable to prediction of breeding values for residual variance than in the case of real data.

An important issue when analyzing field data in a study on genetic heterogeneity of residual variance is that, if the data is not carefully collected for this purpose, it can lead to severe confounding between environmental and genetic effects, which are much more difficult to disentangle than when the focus is on analysis of the mean of a trait [5,6].

We hypothesized that this type of confounding could be more marked in the case of GBW, because this trait is more influenced by maternal effects. Although more sophisticated procedures are probably needed to provide stronger support for the existence of genetic variance of residual variance of beef cattle traits, we found Ev estimates of comparable magnitude for both traits studied.

Yang et al. [25] verified that estimates of genetic effects on the residual variance, and also the genetic correlation between mean and variance, r_mv_, are dependent of the scale on which the data are analyzed under a structural model. However, it is not clear to which extent scale effects influence inferences under the two-step approach. Also, Yang et al. [25] highlighted the importance of confirming that normality assumptions are met when genetic heterogeneity of residual variance is studied. One of the major problems of the two-step approach is that, when residuals are normally distributed, their squares approach a chi-square distribution [6]. Thus, even after log transformation, the distributions of the response variable in the second step are negatively skewed and leptokurtic, which violate the normality assumptions of the linear mixed model and probably explain part of the bias identified for estimates of genetic variance of residual variance in the simulations.

As pointed out in Wilson and Hilferty [26], it is expected that using a cube-root rather than a log transformation of squared residuals produces a response variable with a distribution that is closer to normal. Results of a preliminary simulation suggested that accuracies of sire EBV for residual variance would be higher and variance components will be less biased when using cube-root transformed squared residuals as response in the second step, especially when model HOM is fitted in the first step (data not shown). Future studies could be aimed at comparing these procedures more precisely.

The two-step approach provided some evidence of useful genetic variability on the residual variance of GBW and YW, but important drawbacks were identified. Thus it would be interesting to apply more sophisticated procedures in order to obtain more precise estimates of variance components of residual variance in beef cattle. In this context, structural models implemented in a Bayesian framework are especially appealing, while REML approaches [3,27] are expected to be suitable to study the genetic heterogeneity of residual variance in large populations. In the present study, attempts to fit the double hierarchical generalized linear model proposed by [27] did not converge. Also, direct estimation of r_mv_ and an extension of heteroscedastic models to multi-trait analyses could be the subject of future research in order to optimize selection for both average performance and increased uniformity in beef cattle.

## Conclusions

Results suggest that both genetic and environmental effects influence residual variance of weight gain from birth to weaning and long-yearling in Nellore beef cattle and that uniformity of these traits could be improved by selecting for lower residual variance, when considering a large amount of information to predict genetic merit for this criterion. Stronger statistical support for the action of genetic effects on residual variances was obtained when the model for the mean of each trait accounted for heterogeneous residual variances among sire families. More adequate methods are needed to study the genetic heterogeneity of residual variance in beef cattle.

## Competing interests

The authors declare they have no competing interests.

## Authors’ contributions

HHRN participated in the design of the study, carried out data preparation, simulations and variance component estimation, participated in the analysis of results and drafted the manuscript. RC participated in the design of the study, data preparation and analysis of results and drafted the manuscript. SAQ participated in the design of the study and analysis of results and drafted the manuscript. All authors read and approved the final manuscript.
